# PIIKA 2.5: Enhanced quality control of peptide microarrays for kinome analysis

**DOI:** 10.1371/journal.pone.0257232

**Published:** 2021-09-10

**Authors:** Connor Denomy, Conor Lazarou, Daniel Hogan, Antonio Facciuolo, Erin Scruten, Anthony Kusalik, Scott Napper

**Affiliations:** 1 Department of Computer Science, University of Saskatchewan, Saskatoon, Canada; 2 Vaccine and Infectious Disease Organization (VIDO), Saskatoon, Canada; 3 Department of Biochemistry, Microbiology, and Immunology, University of Saskatchewan, Saskatoon, Canada; Centre de Recherche en Biologie cellulaire de Montpellier, FRANCE

## Abstract

Peptide microarrays consisting of defined phosphorylation target sites are an effective approach for high throughput analysis of cellular kinase (kinome) activity. Kinome peptide arrays are highly customizable and do not require species-specific reagents to measure kinase activity, making them amenable for kinome analysis in any species. Our group developed software, Platform for Integrated, Intelligent Kinome Analysis (PIIKA), to enable more effective extraction of meaningful biological information from kinome peptide array data. A subsequent version, PIIKA2, unveiled new statistical tools and data visualization options. Here we introduce PIIKA 2.5 to provide two essential quality control metrics and a new background correction technique to increase the accuracy and consistency of kinome results. The first metric alerts users to improper spot size and informs them of the need to perform manual resizing to enhance the quality of the raw intensity data. The second metric uses inter-array comparisons to identify outlier arrays that sometimes emerge as a consequence of technical issues. In addition, a new background correction method, background scaling, can sharply reduce spatial biases within a single array in comparison to background subtraction alone. Collectively, the modifications of PIIKA 2.5 enable identification and correction of technical issues inherent to the technology and better facilitate the extraction of meaningful biological information. We show that these metrics demonstrably enhance kinome analysis by identifying low quality data and reducing batch effects, and ultimately improve clustering of treatment groups and enhance reproducibility. The web-based and stand-alone versions of PIIKA 2.5 are freely accessible at via http://saphire.usask.ca.

## Introduction

Kinase-mediated phosphorylation is a central mechanism of signal transduction and regulation of protein activities in eukaryotes. These reversible protein modifications serve as the defining events that immediately precede many cellular responses, represent highly attractive targets for therapeutic intervention [1], and can aberrantly affect cellular signalling resulting in disease [2]. With that, there is increasing priority to define the complete set of kinases and their activity (the kinome) to investigate complex biology, reveal potential therapeutic targets, and identify biomarkers of important phenotypes. This has given rise to a number of technological approaches for defining the kinome [3]. Within these technologies, kinome profiling using peptide arrays provides a robust and versatile method with the decided advantages of not requiring species-specific reagents and being amenable to high-throughput analysis.

In peptide microarrays for kinome analysis, surrogate substrates of kinases are represented by short (15-mer) peptides, each containing the phosphoacceptor site at the central position and maintaining the surrounding amino acid residues as present in the intact protein. Upon exposure to a cellular lysate, the extent to which each peptide is modified through phosphorylation depends upon the activity of the corresponding kinase. By comparing lysates from cells under different biological conditions, it is possible to determine the relative degree of kinase activity, as well as anticipate the relative extent of modification of the proteins represented by the peptides. Peptide arrays have proven a low-cost, versatile tool for kinome profiling. In particular, the opportunity to create customized arrays, coupled with the emergence of software platforms that predict the phosphoproteome of virtually any species, enables a highly versatile option for those looking to incorporate the technology, in particular for research where species-specific reagents are limiting [4].

A key event in the advancement of the technology was the development of software customized for the biological and technological nuances associated with the peptide array data. The Platform for Integrated, Intelligent Kinome Analysis (PIIKA) provides the ability to identify differential phosphorylation events that are responsive to a particular treatment [5]. PIIKA also enables identification of peptides with inconsistencies among the technical replicates on an individual array as well as among biological replicates (e.g., different responses to the same treatment). The PIIKA platform was later expanded upon with PIIKA2, which incorporated addition tools in the categories of cluster analysis, statistical analysis, and data visualization [6]. The peptide array technology, with analysis of the data through the PIIKA/PIIKA2 software platform, has proven effective for investigation of a number of biological processes in a range of species [7–9]. As the technology continues to evolve, a number of recurring unresolved technical issues have been identified that limit the extent to which biology can effectively be extracted from the peptide microarray data: mislabelled pixels during scanning and gridding of the arrays, inconsistency among datasets from apparent technical replicates, and systematic regional differences that exist within the arrays.

To correct for these issues, we developed an upgrade to PIIKA with three major quality control features that provide mechanisms to enhance the extraction of meaningful biological information from kinome data. The first feature provides a metric by which the mislabelling of pixels during scanning, a serious problem with kinome arrays, can be detected. The second feature provides a metric that allows for determination of arrays that are significantly deviant from other arrays in the experiment due to non-intended factors. These arrays have the potential to greatly corrupt downstream analyses, especially analyses in which values are aggregated together. The third feature is a new background correction technique that can reduce spatial bias more greatly than background subtraction, which is the most commonly used method for background correction. The new utilities are a crucial part of an improved kinome analysis pipeline, and their results are provided to the user alongside the regular output from PIIKA. In this paper, we describe the methodology behind, and benefits of, using these new features to address some of the challenges present in kinome arrays. These new features are applied to a well-characterized kinome microarray experiment and shown to improve the final results.

## Materials and methods

### The detection methodology dataset

The utility of the PIIKA 2.5 program was investigated within the context of peripheral blood mononuclear cells stimulated with lipopolysaccharide (LPS), the ligand for Toll-like receptor 4 (TLR4) [10]. This ligand, in this cell population, is a well-characterized biological system, including through kinome analysis [11, 12] and provides an established framework for investigation of data analysis tools [13]. A distinct advantage of this system is the rapid induction of expression and release of tumour necrosis factor alpha in response to LPS stimulation, which provides a readily quantifiable indictor of cell responsiveness [14] as well as the ability to dampen the LPS-induced tumor necrosis factor alpha (TNFa) release through the administration of cortisol [15]. There was a total of 27 arrays in this experiment, representing three biological replicates undergoing three treatments (control, LPS, LPS and cortisol) performed identically on three different days.

### Isolation and stimulation of immune cells

Porcine blood was collected and transferred to 50-mL polypropylene tubes and centrifuged at 1400 × *g* for 30min at 20°C. The experimental protocol was approved by the University of Saskatchewan Animal Care and Use Committee-Livestock (AUP20190084) and sample collection was in accordance with the Canadian Council on Animal Care guidelines. White blood cells were isolated from the buffy coat and mixed with phosphate buffered saline (PBSA) + 0.1% ethylenediaminetetraacetic acid (EDTA) to a final volume of 35mL. The cell suspension was layered onto 15 mL of Ficoll-paque plus (Amersham Biosciences, GH healthcare) and centrifuged at 400 × *g* for 40min at 20°C. Peripheral blood mononuclear cells (PBMC) from the Ficoll-PBSA interface were collected and resuspended in 50ml cold PBSA +0.1% EDTA. The suspension was centrifuged at 1200rpm for 10 minutes at 4°C to wash. The resultant pellet was resuspended in 50ml cold PBSA (no EDTA) and centrifuged at 1200rpm for 10 minutes at 4°C. This wash was repeated again for a total of three washes and pellets from the same animals were combined. Isolated PBMCs were cultured in RPMI medium (GIBCO) supplemented with 10% heat-inactivated fetal bovine serum. Isolated PBMCs were rested overnight prior to stimulation. Purified PBMCs (10×10^6^) were stimulated with 100 ng/mL LPS (*Escherichia coli* 0111:B4) (Sigma-Aldrich), 100ng/mL LPS with 1μM hydrocortisone (added to media 30 minutes before LPS stimulation), or media for 4 hr at 37°C. This quantity and type of LPS was previously shown to induce cellular responses in porcine monocytes. Cells were pelleted and stored at −80°C before use with the peptide arrays.

### TNFa ELISA

Cell supernatants were diluted (1:2) and used for enzyme-linked immunosorbent assays (ELISAs) for porcine tumor necrosis factor alpha (TNFa) as per the R&D Systems DuoSet Development Systems ELISA kit.

### Kinome analysis

Application of kinome arrays was performed using previously described protocols [4]. Arrays were manufactured by JPT Innovative Peptide solutions. Peptides were chosen for the arrays based on known phosphorylation sites relevant to the experiment, as well as other computationally predicted sites using DAPPLE [4]. A total of 27 arrays were analyzed, representing three animals and three different treatments (control, LPS, LPS + cortisol). This was done in triplicate, with each set of replicates being analyzed on different days. Each array includes 282 unique peptides with nine replicates each.

### Detection of inaccurate feature size

**Fig 1:**
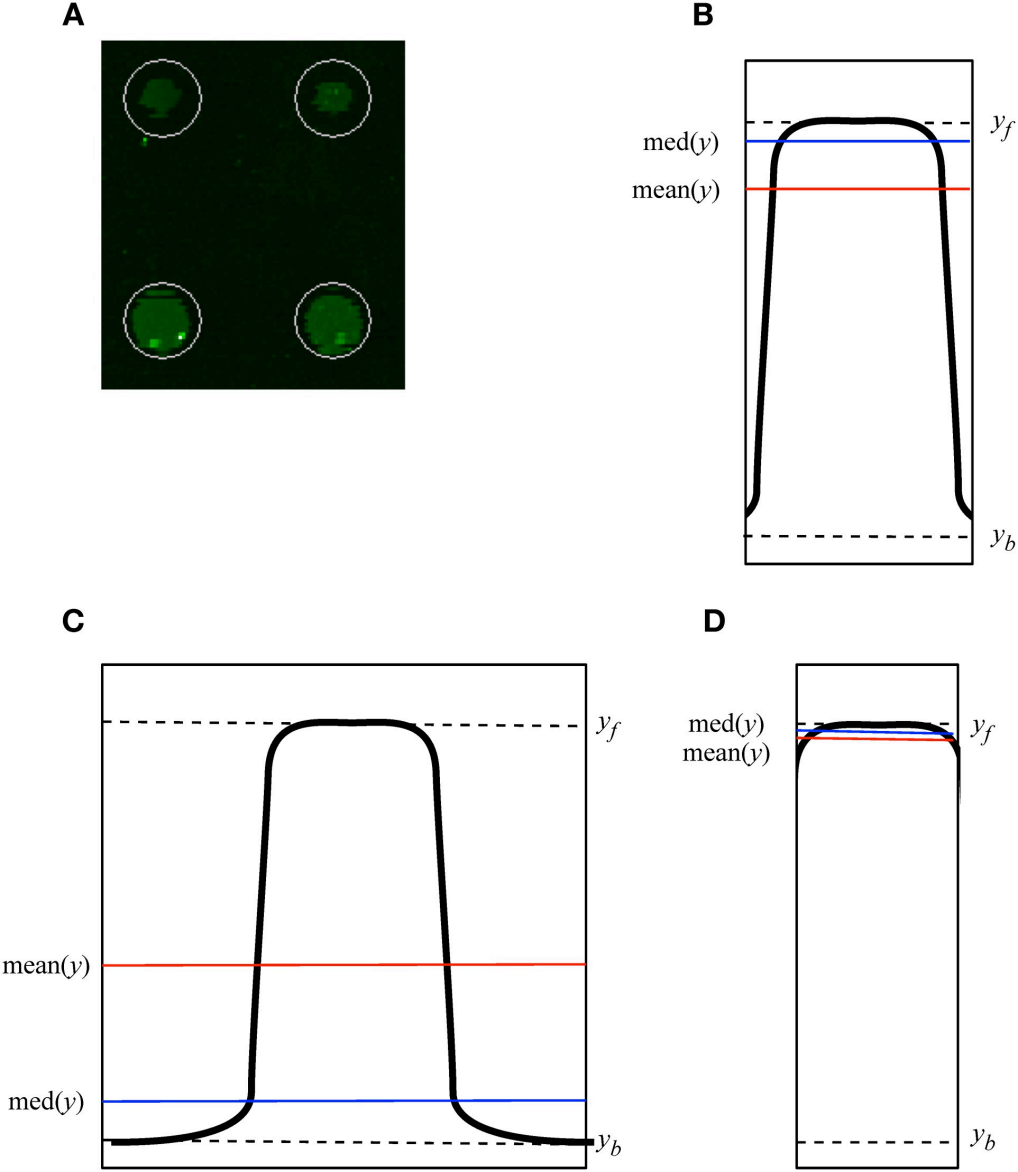
Over-estimation of spot size introduces error in estimation of mean and median spot intensity. A. The automated spot finding provided by the scanner software defaults to using a feature size (shown by the white circle) that captures the entire area of all spots, even the largest spots on the physical array. B. With an accurate feature size values of mean(*y*) and med(*y*) give a true representation of the spot. The vertical edges of the rectangle show the boundary between actual foreground and background pixels constituting the spot. C. Overestimated feature size, showing the resultant reduced mean (mean(*y*)) and further reduced median (med(*y*)). The mean estimated foreground intensity is lowered from the true foreground intensity (*y*_f_), towards the true background intensity (*y*_b_). D. Under-estimating feature size results in increased mean and median, though they are in the correct relationship and are closer to their true values than in panel C. This figure represents a simplification as the rendering in 2 dimensions does not show that the number of pixels increases linearly with the area of a circle, which itself increases as the square of the radius.

**Fig 2:**
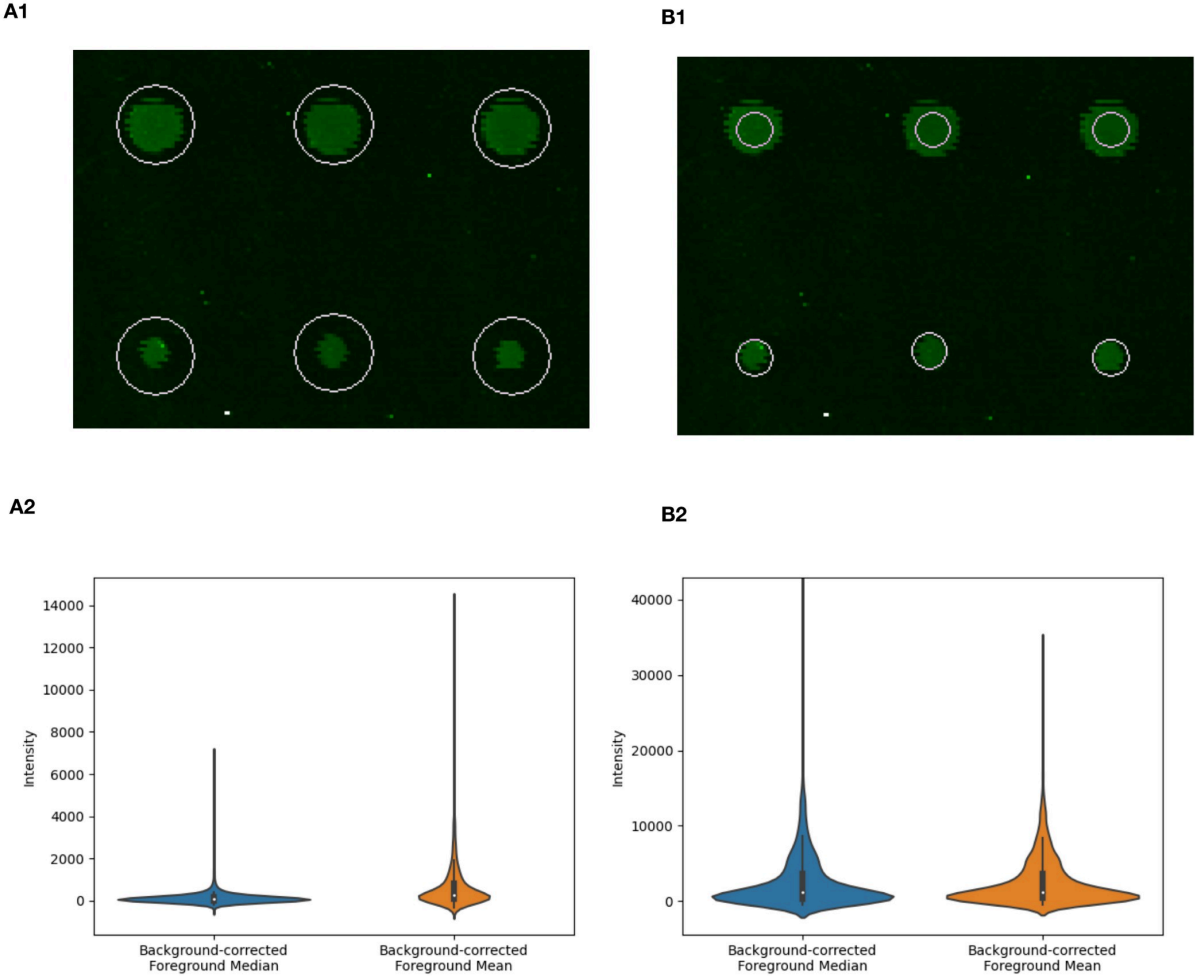
Over-estimation of spot size can affect distribution of spot intensity values: Spots on array from the porcine TLR4 experiment described earlier. Panel A1 shows an over-estimated feature size as a result of the automated procedure provided by the scanner and scanner software, while panel B1 shows the same spots subject to a manually adjusted feature size. Panels A2 and B2 show violin plots of calculated spot intensities for all spots on the array using the corresponding feature size (A1 and B1, respectively). The over-estimated feature size (A1) results in a highly skewed distribution of background-correct median spot intensities and background-corrected mean spot intensities (A2). The effects on the distribution of values includes the range of intensity values, as shown in the y-axis limits shown in Panels A2 and B2. The feature sizes in panels A1 and B1 result in the situations shown in Fig 1C and 1D, respectively.

**Fig 3:**
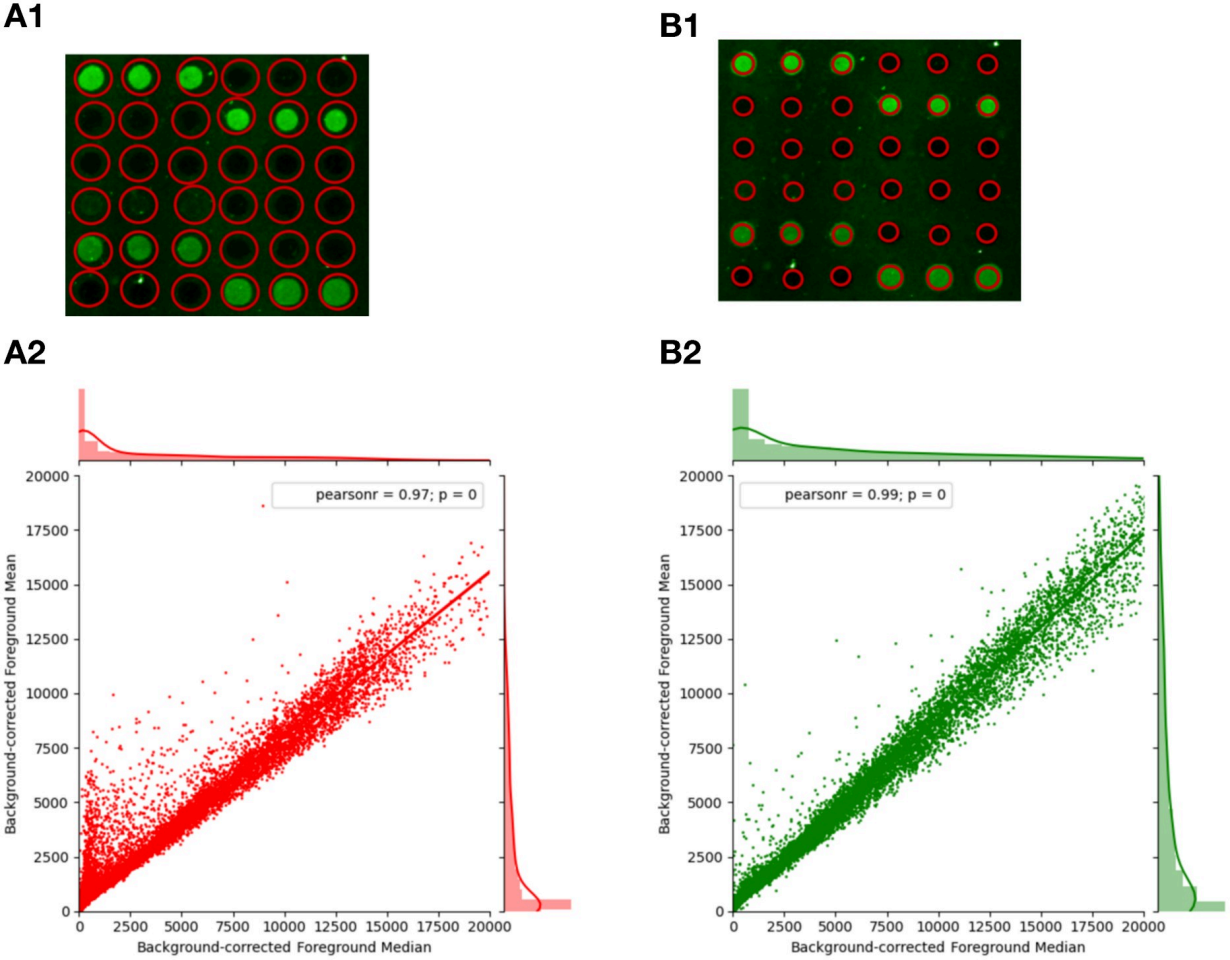
Detection of mislabelled pixels due to errors in spot alignment. A1. Scanning grid where spot size is too large. A2. Linear regression of foreground means (less background) on foreground medians (less background) of a typical suboptimal spot alignment where the estimated spot (feature) size is much larger than the feature’s actual size (shown A1), capturing much of the background area of the array. This leads to the indicative crest of points x < 5000 and a slope much lower than 1 (0.755). B1. More accurate spot size used for scanning. B2. Linear regression of background-corrected foreground means on background-corrected foreground medians of a spot alignment better optimized for kinome microarrays, manually selecting a much smaller diameter for each feature (shown B1). Improved alignment removes the crest feature in the regression plot and raises the slope to 0.855. Frequency histograms along the x- and y-axes show the overall distribution of the background-corrected median and the background-corrected median, respectively. In A1 and B1 actual spots are shown in green, and the boundary of the area labelled as foreground pixels is shown in red. Data is taken from the TLR4 experiment described earlier.

The vendor scanner software first creates a feature of an estimated size around each spot. It defaults to a feature size that will capture even the largest spots on the array (Fig 1). Pixels of the image inside the feature are labelled as foreground and pixels outside the area of the feature are labelled as background. Mislabelling occurs when foreground pixels are labelled as background, or vice versa. This mislabelling can have major effects on the distribution of intensity values obtained from an array (Fig 2) and on downstream analysis. Miscategorized pixels can lead to inaccurate results. In PIIKA 2.5, inaccurate assessment of microarray spot size is detected by utilizing the difference between the median and mean for each spot. Both of these summary statistics are calculated from the intensity values of all foreground pixels deemed to constitute the spot. In cases where the feature size is too large, the median intensity for each spot is lowered (Fig 1C), as mislabelled spots vastly outnumber properly labelled spots as a consequence of the overly large area of the labelling circle. The mean is not as shifted, however, as the intensities of the most intense, properly labelled pixels are much higher than the intensities of mislabelled pixels. This contrasts with the expected median and mean values (Fig 1B). One result of this effect is a skewed distribution of background-corrected mean intensity values and background-corrected median intensity values (Fig 2). Another result is that the slope of the median vs. the mean across the entire array is appreciably lower than one (Fig 3A2). Using this slope as a guideline, we have implemented a three-tiered “stoplight system” to provide feedback to the user. Arrays are given a green, amber, or red “light” depending on the mean/median slope. A slope less than 0.85 (but at least 0.70) is given an amber light, an indication that manual intervention in the operation of the vendor scanner software may be required. A slope less than 0.70 is given the red light, which indicates strong bias that is in urgent need of correction. These values are based on the trends seen across hundreds of in-house arrays but are ultimately arbitrary. More analysis is required to determine alternate slope thresholds. In Fig 3, the data set shown in panel A2 was given a “red light”, while that in B2 was given a “green light”.

### Background scaling

The background scaling process is performed using the data output by the array scanning software, which includes each probe’s foreground and background medians (median of all the pixels deemed to be foreground/background). For each probe, its foreground intensity is the total of the intensities of all pixels deemed to be foreground pixels, and analogously for background intensity. Typically, to compensate for various systematic sources of error related to the location of a spot on the physical array (location bias), foreground intensity is “corrected” by subtracting the background intensity. However, this does not take into account how the local pixel intensities relate to those on the microarray as a whole. Background scaling does this. With background scaling, both intensity values are first divided by the ratio between the local background intensity and the median background intensity of the entire array (Eq 1). This ratio is an additional factor accounting for location bias by considering the relationship between local background pixels (for the spot under consideration) and the background pixels across the entire array. As shown in results, background scaling sharply reduces the location-associated variation between technical replicates.

fs=flblbm-blblbm=flbmbl-bm (1)

**Equation 1**: *f*_*s*_ is the scaled final foreground intensity for the target probe (*f*). *f*_*l*_ and *b*_*l*_ represent the original local foreground and background intensities for *f* and *b*_*m*_ is the median background intensity of the entire array.

### Outlier detection by inter-array comparison

Outlier detection is performed using techniques adapted from the *R* [16] *Bioconductor* [17] packages *arrayQualityMetrics* [18] and *affy* [19]. Outlier arrays are determined by inter-array comparisons using measurements of the mean absolute difference between the data for all corresponding probes between arrays. That is, we calculate the distance between each array pair by determining the mean of the absolute values of the differences between all corresponding probes on the two arrays. There is one such mean or distance for each pair of arrays. Then, for each array, the distances between it and all other arrays in the experiment are collected and totalled. Finally, the total distances for all arrays are subjected to a one-way analysis of variance (ANOVA) and p-values calculated. Arrays that are significantly different past a margin of p < 0.05 according to Tukey’s HSD test, corrected for multiple observations using the Bonferroni correction, are marked as outliers. As in the determination of pixel mislabelling, a “stoplight system” is implemented here as well. Arrays marked as outliers are given a red light and best practice is to remove them entirely. Arrays that trend towards significance (p < 0.1) are given an amber light and could be included in subsequent analysis with caution. Other arrays are given a green light by this analysis. One additional calculation is performed. For each array, the sum of distances to all other arrays (i.e., all its inter-array distances) is also determined. The calculation is part of the ANOVA, but the total can be used to represent the variability of each array with respect to all the others. A threshold value is approximated for the value of the sum of distances at which the ANOVA result has a p-value of 0.05. This threshold is shown in visualizations (Fig 5A).

## Results

In exploring the effects of the three new features of PIIKA 2.5 we consider each in isolation, with remaining stages of analysis as typically performed with PIIKA. This is to ensure that the behaviour observed is a result of the feature under discussion rather than the result of a combination of effects.

### Detection of inaccurate feature size

Automatic alignment of microarray spots as implemented by the GenePix software is often inaccurate on kinome arrays. Using the automatic alignment can cause over-estimation of the size of the spot which in turn causes pixels in the spot that should be labelled as background to be labelled as foreground and included with the foreground in the subsequent calculations of the mean and median (Figs 1 and 2). This mislabelling yields a distortion of data that causes the background-corrected mean and median values to diverge from the true values. The range of intensity values is also reduced with an over-estimated feature size (Fig 2). A more reliable radius of the spot should be smaller, containing less background area. Indeed, it is preferable to make a spot too small as opposed to too large (Fig 1D). As the spot size becomes larger, more background pixels become mislabelled, rapidly leading to the high intensity true foreground pixels becoming outnumbered by the low intensity mislabelled background pixels. This causes the median to shift much lower so that the median point is likely to be the intensity of a mislabelled background pixel, while the mean remains high due to the very high values of the true foreground (Fig 1C). This can skew the distribution of background-corrected mean and median intensity values (Fig 2).

As a simplified example, consider a probe (spot) the size of a single pixel, with a true foreground intensity of 1000 and a background area surrounding it with an intensity of 1. If the spot size is overestimated to include the background pixels, this changes the value of the median foreground intensity all the way to 1, the value of the true background intensity. The mean is also shifted considerably lower, but not as extremely. This effect leads to two distinct features of the data, most easily observed by examining a plot of the background-corrected median and the background-corrected mean (Fig 3). First, the mislabelled points create a region above the linear regression line, with a background-corrected median intensity less than 5000, and a corrected mean intensity between 1000 and 12000. The region resembles a fin. The second feature, caused by the greater lowering of the median than the mean in the poorly aligned probes, is an overall linear regression line with a slope appreciably lower than one. In comparable microarray technologies and other biological distributions this is a highly unusual property.

Based on preliminary empirical study on hundreds of in-house datasets (results not shown), a mean-over-median slope of 0.70 or lower was rarely seen. Therefore, a slope of 0.70 is a reasonable threshold for a dataset to be flagged as having unacceptable levels of bias due to over-estimated spot size and in urgent need of alignment correction. Similarly, a slope under 0.85 but greater than 0.70 is chosen as indicating a potentially problematic dataset that may require either manual alignment or more caution with downstream analysis. Specifically, within the porcine TLR4 signalling dataset, there are no arrays below the 0.70 threshold, but three (11.11%) below 0.85. Realignment of the GenePix grid is the most straightforward way to overcome this bias. These thresholds were not rigorously determined, though based on trends seen through investigation of hundreds of datasets. Future investigation is required to determine thresholds in a more systematic manner.

### Background scaling

Another common source of systematic error in kinome microarrays is an increased intensity (background and foreground) towards one end of an array caused by the physical properties of the array such as orientation. While background subtraction alone is effective at reducing such location biases, the background scaling calculation as described in the Materials and methods further decreases the location-associated variation. Background scaling is similar to background subtraction except that it involves an additional factor that relates local background to the background across the entire array.

**Fig 4:**
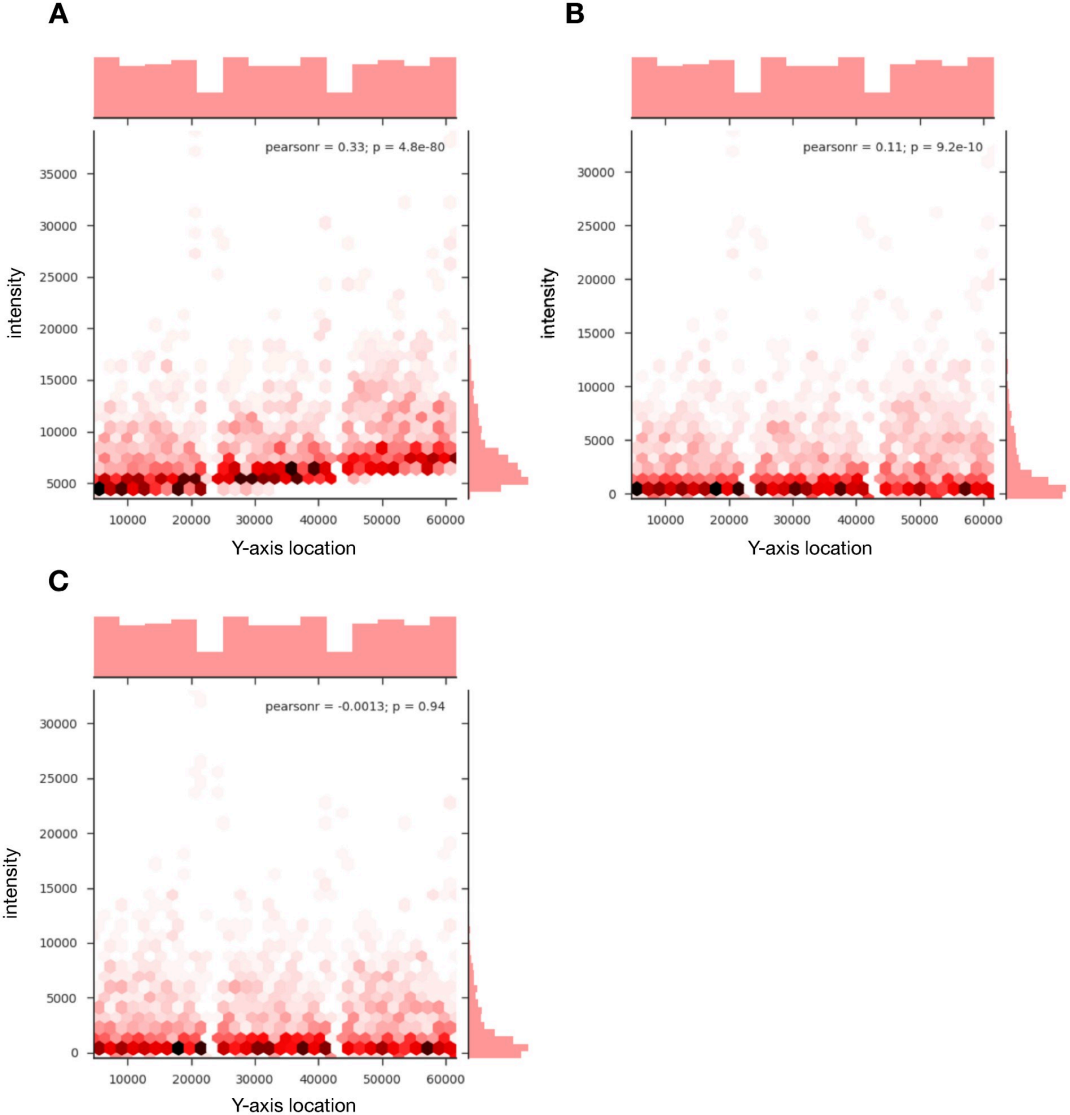
Background scaling to account for regional variations. A. Uncorrected output from an array showing a high degree of bias, creating a correlation between probe foreground intensity and the spot location on the y-axis of the array. B. Output from the same array as A, corrected using background subtraction. The bias has reduced substantially, but there still is a discernible and statistically significant correlation, especially in the probes with higher intensities. C. Output from the same array as A and B, corrected using background scaling, completely removing the correlation between probe intensity and y-axis. Data represents an array taken from the TLR4 experiment described earlier that is neither an outlier nor required any manual alignment correction (mean/median slope > 0.85). Frequency histograms along the x-axis show the distribution of the position of spots on the array (identical for all three panels). Frequency histograms along the y-axis show the distribution of intensities which concentrate at lower values when correction is applied.

The reduction in location bias corrects for inherent location biases present in kinome microarrays to a degree greater than background subtraction (Fig 4). Of the 27 arrays analysed, 74.07% of arrays showed a reduction of the Pearson correlation coefficient between the Y-axis position of a probe and the probe foreground intensity, with the average significance of the correlation decreasing from 0.07 to 0.32 (Table 1). Without correction, this bias is present (p < 0.0001, based on the significance of the Pearson correlation coefficient) in all 27 arrays analyzed in the dataset (Table 1). A binomial test performed on the data reveals a significant effect on the probability of reducing the location bias (p = 0.0066) by using background scaling over background subtraction, though the small sample size of 27 should be taken into consideration when interpreting this result.

**Table 1:** Comparison of different background correction methods on correlation between y-axis location and probe intensity.

Array	Raw		Subtracted		Scaled		Scaling > Subtraction
	Pearson r	p-value	Pearson r	p-value	Pearson r	p-value	
S1 Day 1	0.310	5.10E-69	0.065	4.30E-04	0.0002	0.990	TRUE
S1 Day 2	0.110	1.10E-09	0.025	0.170	-0.0013	0.940	TRUE
S1 Day 3	0.170	5.40E-22	0.034	0.061	0.0130	0.480	TRUE
S2 Day 1	0.230	8.60E-39	0.023	0.200	-0.0320	0.079	FALSE
S2 Day 2	0.110	4.80E-09	0.047	0.010	0.0590	0.001	FALSE
S2 Day 3	0.043	1.80E-02	0.056	0.002	0.0610	0.001	FALSE
S3 Day 1	-0.110	2.60E-09	-0.026	0.150	-0.0027	0.880	TRUE
S3 Day 2	0.330	4.80E-80	0.110	9.20E-10	-0.0013	0.940	TRUE
S3 Day 3	-0.071	9.90E-05	0.039	0.033	0.0710	8.80E-05	FALSE
S4 Day 1	0.240	1.70E-41	0.028	0.13	-0.0350	0.058	FALSE
S4 Day 2	0.190	3.40E-27	0.100	7.40E-09	0.0730	5.70E-05	TRUE
S4 Day 3	0.210	2.80E-31	0.086	2.30E-06	0.0420	0.022	TRUE
S5 Day 1	0.160	3.80E-18	0.024	1.80E-01	-0.0072	6.90E-01	TRUE
S5 Day 2	0.280	1.20E-54	0.130	2.80E-12	0.0650	0.000	TRUE
S5 Day 3	0.170	3.00E-21	0.030	9.50E-02	-0.0110	0.560	TRUE
S6 Day 1	0.370	6.50E-99	0.033	6.90E-02	-0.0320	0.078	TRUE
S6 Day 2	0.330	5.30E-78	0.051	0.0052	-0.0080	0.660	TRUE
S6 Day 3	-0.021	2.40E-01	-0.082	6.60E-06	-0.1000	8.60E-09	FALSE
S7 Day 1	0.330	1.30E-75	0.075	3.70E-05	-0.0090	0.620	TRUE
S7 Day 2	-0.180	1.50E-23	0.060	9.40E-04	0.0370	0.042	TRUE
S7 Day 3	0.090	6.40E-07	0.005	0.790	-0.0280	0.120	FALSE
S8 Day 1	0.260	1.60E-47	0.028	1.20E-01	-0.0210	0.250	TRUE
S8 Day 2	0.240	1.30E-40	0.072	7.50E-05	0.0240	0.180	TRUE
S8 Day 3	0.220	1.30E-34	0.055	2.50E-03	-0.0150	0.400	TRUE
S9 Day 1	0.360	3.70E-94	0.062	6.60E-04	0.0240	0.180	TRUE
S9 Day 2	0.330	1.10E-76	0.059	1.20E-03	0.0150	0.440	TRUE
S9 Day 3	0.270	9.20E-52	0.095	1.90E-07	0.0250	0.170	TRUE
Average	0.212	1.38E-39	0.056	0.001	0.030	0.039	0.741

The table contains 4 major columns, each with a Pearson r-coefficient and a p-value subcolumn. The “Raw” column has no pre-processing applied, using the uncorrected foreground values; the “Subtraction” column uses the typical background subtraction method; and the “Scaling column” uses the new background scaling technique. The fourth column is TRUE if the background scaling outperforms the background subtraction, and FALSE otherwise. The FALSE entries are highlighted in red. Arrays S1-3 are control, S4-6 are LPS and cortisol treated arrays, and S7-9 are LPS treated arrays. For the Pearson r columns, the average is equal to the arithmetic mean of all values in the column. For the p-value, the average is equal to the geometric mean. For the final column, the value in the “average” row is the fraction of arrays in which background scaling outperformed background subtraction.

### Outlier detection by inter-array comparison

**Fig 5:**
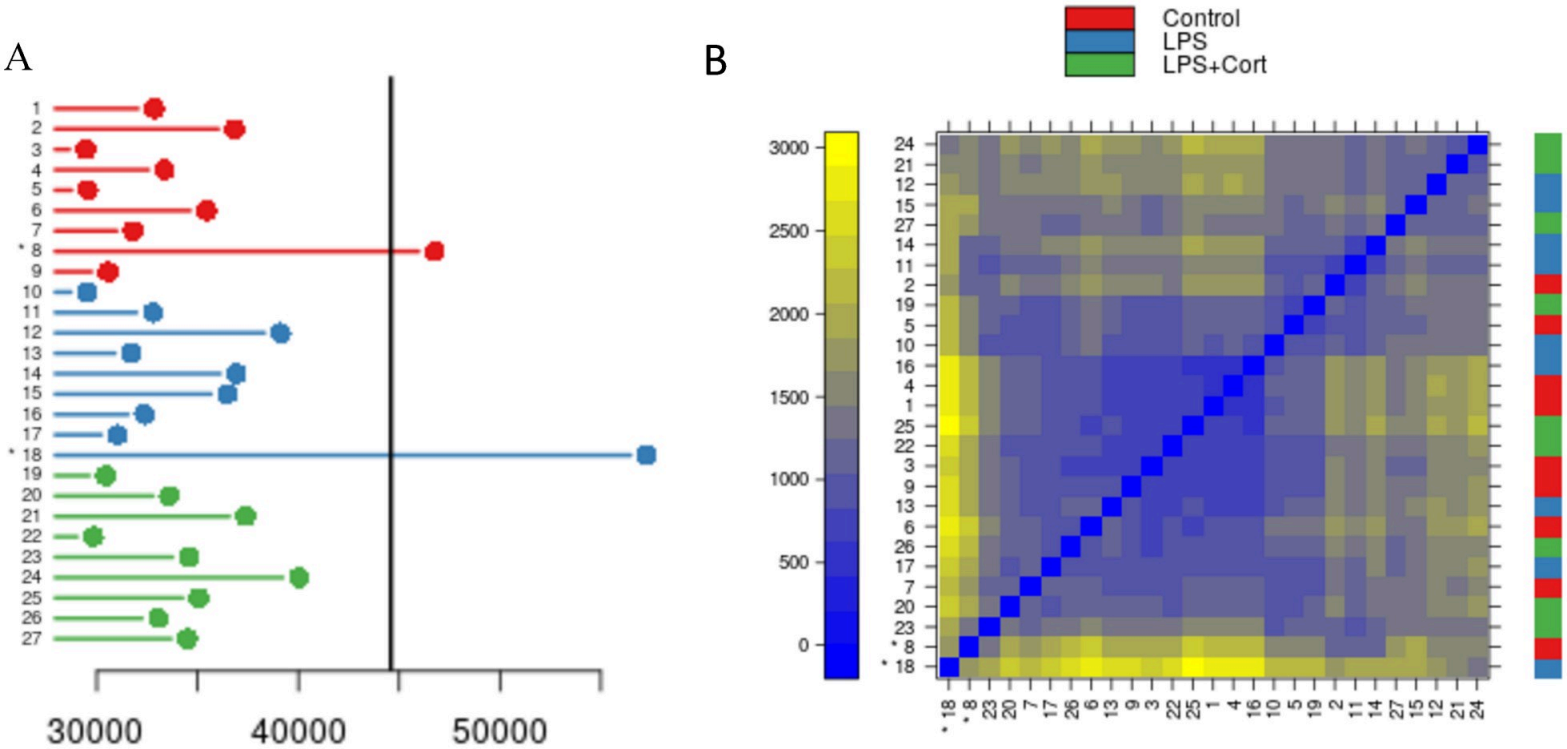
Identification of outliers by inter-array comparison. A. Sum of pairwise distances between the given array and all other arrays (N = 27, consisting of 3 biological replicates with 3 technical replicates each per treatment). The indicator line represents the point where the sum for a given array is significantly greater than the others with a p-value of < 0.05 according to one-way ANOVA with Tukey’s HSD test, corrected for multiple comparisons. Datasets 8 and 18 are outliers with p-values of 0.021 and < 0.001, respectively. B. Heatmap showing pairwise distances between arrays. These are sorted from least significant to most significant difference of sums on the y-axis and the reverse on the x-axis. The color bar on the right indicates the treatment group of the array shown on the x-axis, with red representing control, blue representing LPS alone, and green representing LPS with cortisol. Datasets 8 and 18 are present in the bottom left of the heatmap and are marked by asterisks.

Arrays that are significant outliers from the other arrays in the experiment indicate a systematic error that can have profound consequences on data analysis. This error is not often correlated with intended experimental factors and is largely independent of treatment or biological replicate. Within the twenty-seven datasets under consideration, two arrays, occurring on different experimental days and reflecting different treatment conditions, were flagged as being problematic (Fig 5A). For each pair of arrays, a distance was calculated by summing the mean absolute differences between corresponding probes. Arrays with a significantly high sum of distances (p < 0.05) as determined by one-way ANOVA are flagged as outliers. For each array, the distance between it and all other arrays in the experiment can be summed. By determining the sum corresponding to a p-value of 0.05 from the ANOVA test, a quick visual synopsis of the variation in datasets is possible (Fig 5A). A “stoplight” visualization is used to convey the results to the user, with “green” and “red” indicating “no problem detected” and “outlier detected”, respectively, with “amber” suggesting caution for use of a dataset subsequent analysis. For the dataset from the TLR4 experiment, there are no arrays flagged as “amber” (0.05 < p < 0.1), but with arrays 8 and 18 (p < 0.05) getting a “red light”. All other arrays received a green light.

**Fig 6:**
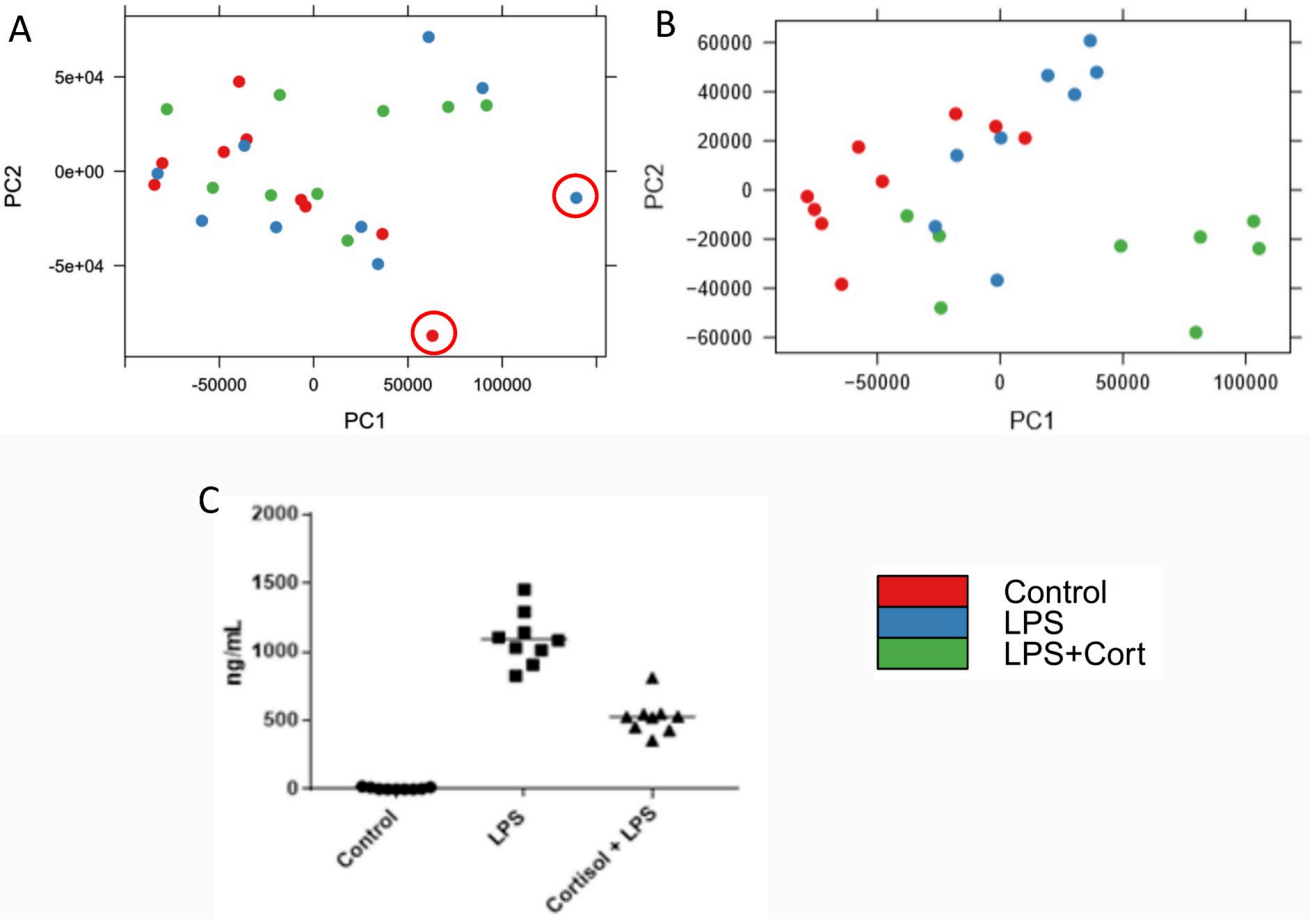
Principal component analysis before (A) and after (B) removal of the arrays determined to be outliers in Fig 5A. The two outliers removed are highlighted in panel A. This data represents 2 treatment groups (blue and green) and one control (red) with 3 biological replicates performed on three different days. C. ELISA data showing differential TNFa levels across the different treatment groups. This shows a strong difference in the immune response, which leads to the expectation that the PCA should show distinct clustering.

While the occurrence of two problematic datasets among twenty-seven might seem inconsequential, it can have substantial impact on the downstream analysis. For example, principal component analysis performed with the inclusion of the red-flagged datasets fails to separate the datasets on the basis of treatment condition (Fig 6A). The same analysis, performed after removal of the two flagged datasets, offers improved separation of the datasets according to treatment groups (Fig 6B). This was confirmed by calculating both the Dunn index and Davis-Bouldin Index (DBI) before and after removal of the outlier arrays. Before outlier removal, the Dunn index and DBI were 0.62 and 0.82 respectively. Following removal, these values were improved to 0.18 (DBI) and 0.97 (Dunn index). The treatment conditions were selected to result in cell populations of distinct phenotypes. In particular, LPS stimulation is known to induce Toll-like receptor signalling that manifests in the induced release of pro-inflammatory cytokines such as TNFa. Within the LPS-stimulated cells there is a clear and consistent response of induced TNFa release (Fig 6C). Cortisol serves to dampen pro-inflammatory responses, including those induced by TLR agonists such as LPS. The pre-treatment of the cells with cortisol results in a clear and consistent reduction of LPS-induced TNFa release (Fig 6C).

## Discussion

Quality control analysis for microarray programs in several popular R packages has led to increased reproducibility [20–22], one of the most important goals in modern high-throughput data analysis. Given the challenges presented by the peptide array technology, it was desirable to have similar quality control filters integrated into PIIKA 2 that specifically targeted major identifiable problems.

One of the most apparent issues in kinome quality control is the lack of a dedicated kinome microarray imaging software. Using automatic grid alignment for the microarrays creates an error caused by mislabelling pixels. One solution is manual realignment of the grid in the imaging software, but this is time-consuming and prone to error. Unless specifically told the proper way to estimate spot size (Fig 1), manual size adjustment can just as easily create the same mislabelling error.

By comparing the mean and the median of each probe after background correction we can get a measurement of the symmetry of the data (Fig 2). In a situation with proper pixel labelling, it is expected that the distributions of the means and medians of probes are roughly equivalent (Fig 2B2), but when the foreground intensity is highly skewed (resulting in the median and mean distributions being dissimilar (Fig 2A2)) it indicates that background pixels were captured within the area labelled as foreground (Fig 1C). This is often easily recognizable by the characteristic fin shape and low mean/median slope (Fig 3), but the effects may be more obscure but still detrimental. The probes that lie in the fin area of the graph (typically between mean 5000–15000 and median < 5000) have been significantly shifted down in both background-corrected foreground mean and background-corrected foreground median. These probes are relevant as, if the data were better conditioned, it could be that these probes would have much higher overall intensities and be placed among the probes with the highest levels of phosphorylation—the “top hits” in a differential phosphorylation analysis.

Background scaling is an important first step in the correction of location biases. We have discovered that in kinome arrays there exists a systematic bias that causes the intensities to increase as the y-axis value increases (Fig 4A). This bias exists uniformly across all 27 arrays analyzed in the TLR4 experiment and is largely ubiquitous across all microarray technologies [23, 24]. This is a result of the reagent used to selectively stain for phosphorylated peptides being increasingly intense towards one end of the array. Location bias is not adequately corrected with background subtraction alone (Fig 4B). In two-color arrays, many different background correction techniques are used, and have been rigorously analyzed [25]. Methods similar to the one presented here have performed extremely well in reduction of false positives associated with location-associated bias without compromising differential expressed genes in two-color arrays [26]. However, in kinome microarrays, there are fewer options for background correction algorithms due to the relative scarcity of kinome microarray analysis software. We have shown that the location-associated variation can be further reduced by scaling the foreground and background with a local estimate of relative background location bias (*b*_*l*_/*b*_*m*_ in Eq 1) prior to the background subtraction (Fig 4C). Our method is presented as an alternative to background subtraction, adding to existing corrections which also outperform background subtraction in reduction of location-associated biases [27]. Calibration probes and other quality control standards which have been previously unsuccessful due the level of noise between technical replicates can be reinvestigated with the application of background scaling. Future efforts can be made to incorporate calibration probes to eliminate a wider variety of biases that can obfuscate the experimental results now that the location-associated variation can be more adequately corrected for.

Pairwise comparison of all the probes on each array is an effective tool for measurement of how similar arrays are. The distance between all corresponding probes between two arrays is measured and the mean of all distances from one array to all other arrays determined. Using an ANOVA, the outliers are determined as arrays with a sum of pairwise distances to all other arrays significantly different from the sum for other arrays. In-house trials have indicated that, in general, arrays that are outliers are variant by a large margin (well beyond the outlier threshold) while the non-outlying arrays are very consistent. Being an outlier therefore indicates presence of a factor that affects all of the probes in the array systematically, as opposed to just a particular subset of probes (the highest intensity probes, for example). There may be situations where the outlier arrays represent a biological effect, so it is necessary for researchers to exercise judgement on how to best proceed. In many cases, however, differentially phosphorylated peptides between treatment groups represent only a small portion of the total number of peptides in the array, so it is more likely that identification as an outlier is caused by sweeping systematic biases.

The quality control results are supplied to the user in a folder with the other data that PIIKA provides, containing the analysis of the two metrics described here. The program supplies figures similar to Figs 3 and 5 that show the median/mean slopes for each array analyzed and the determined outlier arrays. A three-tiered stoplight system has been implemented to further guide researchers in making decisions based on the data provided. This system is ultimately subjective but uses objective data to categorize data into three categories: green (having no observable problems), red (having a large detectable issue), and amber (potentially having some issues) based on the criteria described in the Materials and methods. These colours are integrated into the provided figures and more detailed explanations are offered in an accompanying text file. Ideally, it would be possible to categorize the data into just two categories, but the amount of variation in the data creates sufficient ambiguity to make a binary approach impossible. Green quality assessments mean that there was no problem found with the data at all, giving the user more confidence that their data is reliable. Red assessments are accompanied by a message that strongly suggests some correction to the data or complete elimination is required. There is also a third category, amber, for datasets that are not distinctly “bad” nor distinctly “good”. Researchers could still use these datasets effectively with caution, but should downstream analysis become problematic, a potential source of error has been identified.

In addition to the improved quality control and background scaling, there have also been several other PIIKA improvements of note. Firstly, the old method of inputting data into the PIIKA program required time-consuming work of manually pruning the data, removing many of the microarrays’ built-in control spots. These data points would otherwise make the analysis less accurate, as the points are for the use of scanner calibration and user confirmation and are often read by scanners as either 0 values or extremely high values. The final step of this input process required merging of the array files into a single text file. This process required specific knowledge of the input format, which was not covered in great detail by the existing documentation. This input process has been replaced with an automated one, implemented as a Python script, that performs the same tasks. Users are able to upload a single.*zip* file containing all of their arrays as directly outputted by scanner software, saving time and computational resources. Specific probes in the array files are removed by default, such as ‘printing-buffer’ and ‘blank’, but the user has the capability to alter this list to add or remove rows that are not relevant to the data that they wish to analyse. Backwards compatibility is retained among all new features, and users are still able to input experimental results as they were before.

Despite this being a major improvement to the existing kinome microarray analysis pipeline, there are still challenges that have yet to be addressed. The lack of image analysis software specifically tailored for kinome microarrays is a major limitation and addressing this would mitigate many of the problems that are corrected by this update. A dedicated software suite would also alleviate much of the time-consuming labour involved in manual alignment of the array image with the data grid. While background scaling does provide correction for spatial biases, the update to PIIKA does not correct for other systematic biases that we have determined to be evident in kinome microarrays—it only identifies them. It is feasible that eventually problems such as misalignment of the imaging grid, batch bias, and overall array quality will not only be identifiable, but also correctable. Regardless of this, the identification of the problems and their sources is the essential first step of correction. The update to PIIKA provides objective information for researchers to make subjective decisions about their data, which will lead to increased accuracy and technical reproducibility.

PIIKA 2.5 is available in two forms: a web-based version, and a local version that can be installed on the user’s computer. Both versions are available through the Saskatchewan PHosphorylation Internet REsource (SAPHIRE) website at http://saphire.usask.ca. PIIKA 2.5 is free for academic use; users interested in PIIKA 2.5 for commercial purposes should contact the authors.

## Supporting information

S1 File Zip folder containing GPR files. All arrays used in this experiment are available as txt files in the GPR format contained within a single zip folder. Individual files are named as in Table 1, where the first number, which follows S, represents the identifier for the samples. Samples 1 2, and 3 are control, 4,5 and 6 are treated with LPS, and 7,8, and 9 are treated with both LPS and cortisol. The number following this represents the day of experiment. For example, S1_2.txt contains the data for sample number 1 from the experiment performed on day 2.
(ZIP)
